# Impact of Bottle Aging on the Composition and Sensory Properties of Flavored Chardonnay and Shiraz Wines

**DOI:** 10.3390/foods9091208

**Published:** 2020-09-01

**Authors:** Yaelle Saltman, Julie A. Culbert, Trent E. Johnson, Renata Ristic, Kerry L. Wilkinson, Susan E. P. Bastian

**Affiliations:** School of Agriculture, Food and Wine, Waite Campus, The University of Adelaide, PMB 1, Glen Osmond, SA 5064, Australia; yaelle.saltman@adelaide.edu.au (Y.S.); julie.culbert@adelaide.edu.au (J.A.C.); trent.johnson@adelaide.edu.au (T.E.J.); renata.ristic@adelaide.edu.au (R.R.); sue.bastian@adelaide.edu.au (S.E.P.B.)

**Keywords:** bottle aging, descriptive analysis, flavor additives, GC-MS, wine, wine product

## Abstract

Natural flavorings could potentially be used to enhance the intensity of wine aroma and flavor; albeit since flavor additives are not legally permitted winemaking aids, flavored wines would need to be labeled as wine products. In this study, changes in the composition and sensory profiles of flavored Chardonnay (*n =* 2) and Shiraz (*n =* 2) wines were compared at bottling, and then again after 12 months of bottle aging. Flavorings and flavored wines were also analyzed by gas chromatography-mass spectrometry (GC-MS) to determine the key constituents responsible for changes to aroma and flavor profiles. However, many of the volatile compounds identified in flavor additives were not detected at appreciably higher concentrations in flavored wines, which was attributed to the very small quantities of flavorings that were added to base wines. The sensory profiles of control and flavored wines were determined by descriptive analysis, and the addition of flavorings to base wines significantly influenced the perception of some sensory attributes. Flavored Chardonnay wines exhibited enhanced fruit aromas and flavors, while fruit and developed attributes were enhanced in flavored Shiraz wines. Differences in sensory profiles were less apparent in Chardonnay wines following bottle aging, but depending on the flavorings added, flavored Shiraz wines could still be discriminated from their corresponding control wines after bottle aging. Results from this study demonstrate the potential for flavor additives to be used to enhance desirable attributes and/or mitigate wine sensory deficiencies.

## 1. Introduction

Aroma and flavor intensity are important indicators of wine quality, and can be attributed to the presence of volatile compounds derived from grapes, primary and secondary fermentation, maturation and/or aging [1,2]. Whereas, flavor additives can be used to moderate aroma and flavor intensity in food and beverage production [3], regulations governing the use of additives and processing aids in most wine-producing countries prevent their use during winemaking. In Australia, the addition of flavorings to wine renders it a ‘wine product’, according to the Australian and New Zealand Food Standard Code [4]. However, research suggests a significant proportion of consumers (up to 50%) do not understand the meaning of this term, and they may, therefore, be misled by ‘wine product’ labeling [5]. Whilst it is unlikely that flavor additives would ever be used in the production of premium quality wines, they might offer winemakers the ability to moderate the sensory properties of commodity wines or address wines with sensory deficiencies. For example, flavorings could be used to: (i) Enhance the aroma and flavor intensity of wines affected by adverse weather conditions; (ii) mask undesirable ‘green’ (unripe) characters; or (iii) introduce oak-related characters to wine, without the investment in time or capital associated with traditional barrel maturation. Recent studies have, therefore, explored consumer attitudes towards the addition of flavorings to wine [6] and the potential for flavor additives to influence the sensory profiles and consumer acceptance of wine [7].

An online survey (with 1031 participants) concerning attitudes towards the use of additives in food and wine production found Australian wine consumers were generally more accepting of the addition of natural flavorings to wine, than many of the (legally permitted) additives currently used in winemaking (e.g., tartaric acid and sulfur dioxide) [6]. A subsequent study evaluated consumer liking of control (unflavored) and flavored Chardonnay and Shiraz wines, and identified consumer segments who accepted, and in some cases preferred, the flavored wines [7]. The flavorings added to wines were chosen according to findings from previous studies on wine consumers’ flavor preferences [8,9], with flavor combinations optimized via bench-top trials, then refined using feedback obtained from consumers via focus panels [7].

The composition of wine evolves with time, as a consequence of chemical transformations that occur after bottling [10]. These compositional changes can be either desirable or undesirable. For example, hydrolysis of esters during aging of wine can result in the loss of varietal expression, i.e., a decrease in the intensity of fruity, floral characters [11,12], while the formation of phenylacetaldehyde and methional, due to oxidative effects can give rise to over-ripe fruit or cooked vegetable notes [13]. In other cases, pleasant toasty, biscuit, honey, nutty, and/or toffee characters may arise with the aging of white wine [14,15,16]. Similarly, temporal changes in the volatile compounds present in red wines can result in primary fruit characters giving way to caramel, savory, truffle, leather, chocolate, cedar, and/or coffee developed notes [17,18]. Aging of red wine is also associated with modifications in the wine color and mouthfeel properties, due to reactions of polyphenolic compounds [19]. Whereas, some wine styles benefit from extended aging in the bottle, many inexpensive (commodity) wines are intended to be consumed soon after bottling, when varietal expression is at its peak. The stability of flavor additives in the acidic wine medium has not yet been investigated, but the sensory perception of flavorings would be expected to diminish with time. As such, this study sought to determine the impact of bottle aging on the composition and sensory profiles of flavored wines, using a combination of gas chromatography-mass spectrometry (GC-MS) and descriptive sensory analysis.

## 2. Materials and Methods 

### 2.1. Flavorings and Reagents

Flavor additives were sourced from the Product Makers Pty. Ltd. (Melbourne, VIC, Australia; chocolate1039, cinnamon1525, custard1989, orange1883, raspberry228 and vanilla1729) and FlavorSense Corporation (San Rafael, CA, USA; apricotWW3, berry8819, butter10-1206, honey, oak and passion fruit77116). Analytical grade reagents, solvents, and standards used in GC-MS analysis were purchased from Sigma Aldrich (Castle Hill, NSW, Australia), CDN Isotopes (Pointe-Claire, QC, Canada) and Chem-Supply (Gillman, SA, Australia). 

### 2.2. Preparation and Aging of Wines

The preparation of flavored wines has previously been reported in full [7]. Briefly, four inexpensive commercial wines (retailing at ≤ AUD$10 per 750 mL bottle), two 2011 Chardonnay wines from the Riverland and South Eastern Australia (hereafter CH1 and CH2) and two 2011 Shiraz wines, both from South Eastern Australia (hereafter SH1 and SH2), were sourced from Australian wineries and spiked with different combinations of flavorings (Table S1) to generate two flavored versions of each wine (on a 20 L scale). Flavor combinations were selected and optimized based on consumer surveys, bench-top trials, and focus panels described previously [6,7]. Control and flavored wines were then bottled (in 375 mL dark green colored glass bottles) under metal screwcap closures, with minimal ullage (<1–2 mL) and carbon dioxide blanketing, and cellared (in an upright position), in darkness at 15 °C. 

### 2.3. Basic Wine Composition

The pH, titratable acidity (TA, as g/L of tartaric acid) alcohol (% *v/v*), residual sugar (as g/L of glucose and fructose) and volatile acidity (VA, as g/L of acetic acid) of wines were measured (in duplicate) according to published methodology [20]. Results from chemical analyses performed five weeks after bottling (hereafter ‘*t* = 0’) were reported previously [7]; in the current study, analyses were repeated following 12 months bottle aging of wines (hereafter ‘*t* = 1’).

### 2.4. Volatile Composition of Flavorings and Wines

#### 2.4.1. Sample Preparation

For analysis of flavorings, flavor additives (2–3 drops, approx. 0.1 g) were added to 20 mL screw-cap autosampler vials (Sigma Aldrich), together with Milli-Q water (5 mL) and sodium chloride (2.0 g). Vials were sealed and thoroughly mixed with a vortex mixer prior to GC-MS analysis. For analysis of flavored wines, wine (0.5 mL) was placed in a 20 mL screw-cap autosampler vial containing sodium chloride (2.0 g) and Milli-Q water (4.5 mL) and 2-octanol (10 µL, 50 mg/L in ethanol) added as an internal standard. Vials were sealed and thoroughly mixed using a vortex mixer prior to GC-MS analysis. Flavorings were analyzed five weeks after bottling (i.e., at *t* = 0), whereas wines were analyzed five weeks after bottling, then again following 12 months of bottle aging (i.e., at *t* = 0 and *t* = 1). 

#### 2.4.2. GC-MS Instrumentation

Samples were analyzed by gas chromatography-mass spectrometry (GC-MS), with a 7890A GC coupled to a 5975C inert XL mass selective detector (Agilent Technologies, Santa Clara, CA, USA) and equipped with a Gerstel MPS2 Multipurpose autosampler (Gerstel, Mülheim an der Ruhr, Germany). Instrument control and data analysis were performed with Agilent ChemStation software (E.02.02.1431, Agilent Technologies, Santa Clara, CA, USA) and Gerstel MASter software (version 1.3, Lasersan Australiasia Pty. Ltd., Robina, QLD, Australia). Samples were incubated with agitation for 10 min at 50 °C, prior to headspace solid-phase micro-extraction (HS-SPME) for 30 min at 50 °C (with agitation) using a Supelco 50/30 µm divinylbenzene/carboxen/polydimethylsiloxane 1 cm SPME fiber. The SPME fiber was desorbed in the GC inlet containing an ultra-inert glass SPME liner (straight taper with 0.75 mm i.d.), operating in splitless mode at a temperature of 240 °C. The SPME fiber remained in the inlet for 10 min, but with a purge, flow to split vent of 20 mL/min after 3 min. Separation of volatile compounds was achieved using an Agilent J&W DB-WAX capillary column (60 m × 0.25 mm i.d. × 0.25 µm) with ultrapure helium (Coregas, Cavan, SA, Australia) as the carrier gas at a constant flow rate of 1.5 mL/min. The oven program was as follows: 40 °C (held for 5 min), increased to 210 °C at 2 °C/min (held for 5 min), and then to 240 °C at 5 °C/min (held for 10 min), giving a total runtime of 111 min. The MS was operated using positive ion electron impact at 70 eV in either full scan mode (*m/z* 35–350) or select ion monitoring (SIM), with MS source and quad temperatures of 230 °C and 150 °C, respectively. The MS transfer line was held at 240 °C. SIM parameters were as follows: Group 1 (Start time 0.00 min) *m/z* 43.1, 70.1, 71.1, 86.0, 88.1, 101.1 and 116.1; Group 2 (start time 18.01 min) *m/z* 68.1, 79.1, 93.0 and 136.1; Group 3 (start time 30.00 min) *m/z* 39.1, 41.1, 55.1, 57.1, 67.1, 70.1, 71.1, 82.1, 83.1, 84.1, 89.1, 93.1, 95.0, 96.0, 105.0, 106.0, 121.1, 129.1 and 136.1; Group 4 (start time 48.00 min) *m/z* 59.1, 65.1, 69.1, 91.1, 93.1, 104.1, 121.1, 123.1, 136.1, 138.1, 156.1, 163.0, 164.1 and 192.1; Group 5 (start time of 62.00 min) *m/z* 43.1, 55.1, 57.1, 65.1, 77.1, 85.0, 91.1, 92.1, 93.1, 103.1, 104.1, 121.1, 122.1, 128.1, 131.1, 132.1, 135.1, 136.1, 147.1, 176.1, 177.1, and 192.1. Ions in groups 1 and 2 had a dwell time of 100 ms, while those in groups 3, 4, and 5 had a dwell time of 50 ms. Compound identification was achieved using the National Institute of Standards and Technology (NIST) 05 Mass Spectral library database and by comparing retention times and mass spectra with those of reference standards (Table S2), when available. Compound peak areas were corrected relative to 2-octanol. 

### 2.5. Sensory Analysis of Wines

The sensory profiles of control and flavored wines were determined by descriptive analysis (DA). The sensory analyses performed five weeks after bottling (i.e., at ‘*t* = 0’) have previously been described in full [7]; in the current study, analyses were repeated following 12 months bottle aging of wines (i.e., at ‘*t* = 1’). 

A DA panel comprising twelve panelists (seven females and five males, aged between 22 and 60 years), all of whom had previous DA experience and six of whom participated in DA at *t* = 0, was assembled. Panelists underwent five training sessions (1 × 2 h session per week, held over five consecutive weeks). During training sessions, the panel evaluated the aroma, flavor, taste, and mouthfeel attributes of wines, according to standard DA protocol [21], and were introduced to the tasting booths in which formal evaluations would be held (i.e., under controlled ventilation, light conditions, and temperature, being 22–23 °C). The panel generated twelve aroma, seven flavor, and five taste and mouthfeel descriptors for Chardonnay wines and eleven aroma, eight flavor, and five taste and mouthfeel descriptors for Shiraz wines (Table S3); these included the same aroma, flavor, taste, and mouthfeel attributes used for DA of wines at *t* = 0, but several additional descriptors were generated for the bottle-aged wines. Reference standards were developed during early training sessions and were freshly prepared (in covered, opaque black glasses) for use at subsequent training sessions and throughout formal evaluations. Examples of taste and mouthfeel attributes (from low to high) were also provided and comprised creaminess (low-fat milk to full cream milk), acidity (base wine spiked with 0.5 to 2 g/L tartaric acid), bitterness (base wine spiked with 5 to 20 mg/L quinine sulfate), and astringency (felt material to sandpaper). The aftertaste was defined as the length of time for which fruit and/or phenolic attributes were perceived after expectoration. 

During training, panelists practiced rating the intensity of each descriptor, and their performance was evaluated using SENPAQ (version 5.01, Qi Statistics, Reading, UK). Further training was provided to panelists for any attributes with the significant judge by sample interactions. Panel performance was considered to be satisfactory once interactions were minimized, after which, formal evaluations commenced. Four formal evaluation sessions were held (two each for Chardonnay and Shiraz wines), with nine wines presented per session, such that three replicates of each wine were assessed. Wines (30 mL) were assigned random three-digit codes and served in XL5 (ISO standard) 215 mL wine glasses covered with plastic lids, using a randomized presentation order, with wines presented in brackets of four or five samples. Chardonnay wines were served at 14–16 °C and Shiraz wines were served at 22–24 °C. Panelists evaluated wines and recorded the intensity of each sensory attribute using FIZZ data acquisition software (Version 2.47b, Biosystèms, Couternon, France) on 15 cm unstructured line scales with anchor points of ‘low’ and ‘high’ placed at 0% and 100% on the scale, respectively. Between samples, panelists cleansed their palate with filtered water and unsalted crackers during a one min break. Panelists were required to have five min breaks after each bracket. 

### 2.6. Data Analysis

Sensory data were analyzed using a mixed model analysis of variance (ANOVA) with wine sample and replicate as fixed factors and panelists as random factors, including two-way interactions (Tables S4–S7). Fisher’s least significant difference (LSD) was applied as post-hoc comparison *p* < 0.05. Data analyses were performed with XLSTAT (version 2020.1., Addinsoft, New York, NY, USA).

### 2.7. Ethical Statement 

DA panelists gave informed consent before participating in the study, which was approved by the Human Research Ethics Committee of The University of Adelaide (Project No. H-174-2011).

## 3. Results and Discussion

### 3.1. Influence of Flavoring and Aging on Basic Wine Composition

The pH, TA, alcohol, residual sugar, and VA of control and flavored wines were measured after 12 months of bottle aging (*t* = 1), to investigate compositional differences amongst wines attributable to the addition of natural flavorings (Table S8). As expected, compositional differences were observed between the four wines, but no significant differences were observed between control wines and their corresponding flavored wines. Nor were there significant differences between wines after bottling and bottle aging (i.e., at *t* = 0 and *t* = 1, data not shown). Neither the addition of natural flavorings nor bottle aging significantly influenced the basic wine parameters that were measured. 

### 3.2. Volatile Composition of Flavor Additives and Flavored Wines 

**Figure 1:**
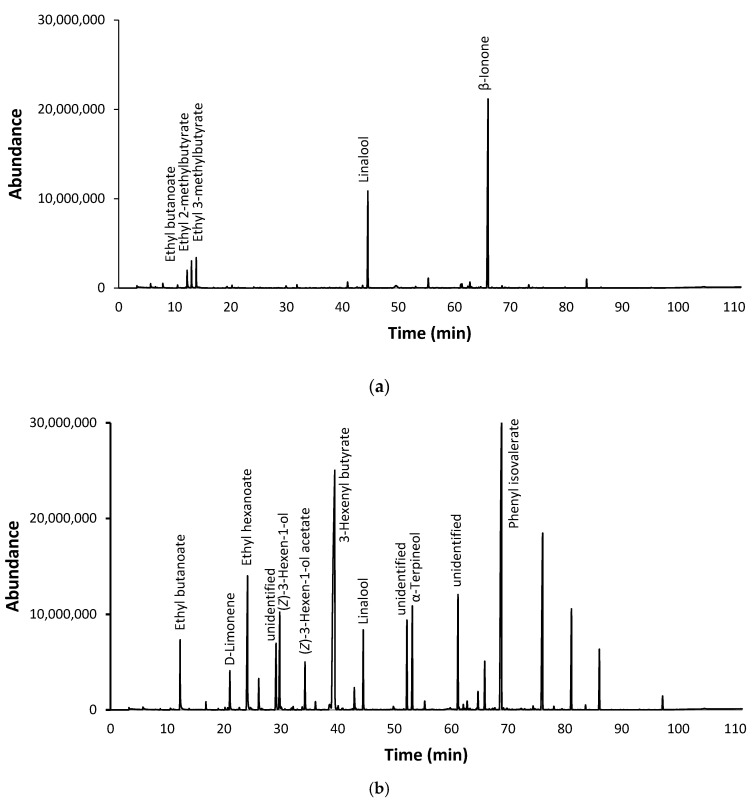
Chromatograms of (**a**) raspberry and (**b**) passion fruit flavor additives.

The composition of flavor additives was analyzed by GC-MS in an attempt to identify the key volatile compounds responsible for their characteristic aromas and flavors. The complexity of flavorings varied considerably, with some flavor additives comprising relatively few volatile compounds, e.g., the raspberry flavor additive (Figure 1a), while others contained an array of constituents; around 20, in the case of the passion fruit flavoring (Figure 1b). The most abundant flavoring constituents were isoprenoids, furans, esters, alcohols and volatile phenols (Table 1), all of which have previously been identified as constituents of grapes and/or wine [22,23,24,25].

Control and flavored wines were also analyzed by GC-MS (at both *t* = 0 and *t* = 1), to determine compositional changes attributable to the addition of flavorings and/or bottle aging. However, flavorings were added to wines in such small quantities, i.e., as 1% solutions prepared from ≤3.0 g/L standards of flavor additives (Table S1), that many of the volatile compounds identified as constituents of flavor additives were either not detected in flavored wines or were present at similar concentrations to those of corresponding control wines (data not shown); irrespective of whether samples were analyzed using full scan mode or following the development of SIM methods to improve selectivity and sensitivity. However, there were some notable exceptions (Table 2). 

Similar levels of *cis*-3-hexenyl butyrate were found in CH1 and CH1 + PF at *t* = 0, but almost 30-fold higher concentrations were observed in CH1 + PF, than in CH1, at *t* = 1. Comparable results were obtained following the addition of passion fruit flavoring to CH2; approximately 50-fold higher *cis*-3-hexenyl butyrate concentrations were found in CH2 + PF, than in CH2 at *t* = 1. Although similar levels of linalool were found in CH1 and CH1 + PF at *t* = 0, CH1+PF contained approximately double the linalool content of CH1 at *t* = 1. The linalool and limonene concentrations of SH1 + C and SH1 + R were similarly found to increase (relative to SH1) following bottle aging. Significant quantities of 2-ethyl hexanol were detected in all control and flavored SH1 wines; levels were higher in SH1 + C than SH1 at *t* = 0, but lower in SH1 + C (than SH1) at *t* = 1. The addition of berry flavoring to SH2 resulted in significantly higher concentrations of linalool and α- and β-ionone in SH2 + B (approximately 55%, 1900% and 360% higher levels, respectively, at *t* = 0). Linalool levels remained similar for control and flavored SH2 wines following bottle aging, but the α- and β-ionone content of SH2 + B increased (by an additional 50–100%) during bottle aging. Approximately two-fold higher concentrations of phenethyl acetate were found in CH2 + H than in CH2. In some instances, compositional differences between control and flavored wines were directly attributable to the addition of flavor additives, but changes observed after bottle aging likely reflect chemical transformations of wine and/or flavor constituents [10]. 

The detection of volatile compounds derived from flavor additives could be improved through various method development strategies, for example, through the extraction of larger volumes of flavored wine, different sampling methods, and/or the use of more specific standards (i.e., isotopically labeled internal standards). In this study, the impact of flavor addition and bottle aging was instead assessed via sensory analysis. However, it should be acknowledged that challenges associated with detecting flavor constituents in wine could have implications for policing the use of flavor additives by industry; i.e., where their use is legally prohibited, flavorings can seemingly impact wine aroma and flavor at concentrations that cannot be readily detected.

### 3.3. Sensory Profiles of Control and Flavored Wines

Results from descriptive analysis of control and flavored Chardonnay and Shiraz wines performed at *t* = 0 and *t* = 1 were compared to determine the impact of flavor addition and bottle aging on wine sensory profiles. As previously reported, the addition of flavorings influenced the sensory profiles of Chardonnay and Shiraz wines [7], but bottle aging also influenced wine aroma, flavor, and/or mouthfeel attributes. The addition of apricot and passion fruit flavorings to CH1 enhanced the intensity of selected fruit and/or floral characters, and diminished the perception of astringency (Table 3). However, after 12 months of bottle aging, differences between control and flavored wines were less apparent; only melon aroma and caramel-lolly flavor were found to be significantly higher in flavored CH1 wines at *t* = 1. The intensity of other sensory attributes increased for CH1, CH1 + A, and CH1 + PF alike. This likely reflects the development of some complexity, due to aging, i.e., increases in the intensity of vanilla, butter, mixed spice, caramel, and oak characters, as well as the occurrence of dried fruit, toast and green notes (Table 3). However, it should be acknowledged that this could also reflect differences in the composition and/or performance of the DA panels between *t* = 0 and *t* = 1. LSD values were higher at *t* = 1 compared to *t* = 0 (data not shown), despite each panel undergoing training. Increased LSD values might also reflect the DA panel’s broader use of the intensity scales at *t* = 1. 

Less favorable results were achieved following the addition of flavor additives to Chardonnay 2 (Table 4). The honey flavoring had little impact on wine aroma or flavor; the honey aroma was not significantly enhanced, and only the perception of oak flavor increased. The addition of passion fruit flavoring surprisingly resulted in less intense fruit characters (including passion fruit aroma), while bitterness, acidity, and astringency were perceived to be more prominent. This may reflect cross-modal interactions [26], as previous studies have shown certain aromas can enhance taste perceptions without directly imparting taste properties [27,28,29]. Certainly, the enhanced acidity perceived in CH2+PF at *t* = 0 was not indicative of any differences in pH or TA (data not shown). 

Bottle aging of CH2 gave similar outcomes to those observed for CH1; i.e., more intense vanilla, butter, orange blossom, mixed spice, caramel-lolly, and oak characters, together with dried fruit, toast and green vegetable notes (Table 4). Interestingly, the bitterness, acidity, and astringency observed in CH2 + PF at *t* = 0 were no longer prominent after bottle aging. At *t* = 1, control and flavored CH2 wines had quite similar sensory profiles, albeit CH2 + H exhibited more intense honey and oak notes, than CH2 (at *t* = 1). Again, the emergence of additional attributes, dried fruit and toast, in particular (Table 4), was consistent with the developed notes associated with bottle age in white wine.

The addition of flavorings to SH1 significantly increased the perception of confectionary and chocolate-vanilla characters, and diminished the earthy aroma of SH1 + R, but there were few statistically significant sensory differences between SH1 and SH1 + C (Table 5). This was surprising given the chocolate flavoring was intended to enhance chocolate notes, i.e., to mimic oak characters. In the case of SH1 + R, sensory differences were attributed to the butter and custard flavor additives present in the raspberry flavoring. This combination of flavors (i.e., butter, orange, custard, and raspberry) seemingly maintained its influence on wine aroma and flavor during bottle aging, and the intensity of confectionary and chocolate-vanilla characters were still significantly different from SH1 at *t* = 1. Again, additional attributes were observed at *t* = 1, i.e., plum, licorice and dried herb aromas, and cherry and green vegetable flavors, possibly due to aging (Table 5). The sensory profiles of SH1 and SH1 + C were still similar. These results suggest the raspberry flavoring that was added to SH1 had greater persistence than any of the flavorings added to the Chardonnay base wines.

The addition of berry flavoring, which comprised berry, custard, and butter flavor additives, to SH2 enhanced the perception of confectionary flavor and diminished the perceived intensity of oak flavor at *t* = 0 (Table 6). After bottle aging, the intensity of red berry and confectionary aromas and chocolate-vanilla flavor were still significantly higher than in SH2. In contrast, the raspberry flavoring did not significantly influence wine aroma or flavor, at either *t* = 0 or *t* = 1. This suggests a higher dose of flavor additives might have been needed to modify wine sensory properties. 

A key aim of this study was to determine the impact of bottle aging on the sensory profiles of flavored Chardonnay and Shiraz wines, and the persistence of sensory qualities imparted by flavorings. In some, but not all cases, the addition of flavor additives did modify the perception of wine aroma and/or flavor. Where flavorings had limited impact on wine sensory profiles, the addition of more concentrated flavorings, or flavorings comprised of different combinations of flavor additives, might achieve more apparent sensory outcomes. Regardless, bottle aging seemingly influenced flavored Chardonnay and Shiraz wines differently. The differences observed between the sensory profiles of control and flavored Chardonnay wines after bottling (i.e., at *t* = 0) were not as apparent after 12 months bottle aging (i.e., at *t* = 1); which may have reflected the development of secondary vanilla, butter, spice, caramel and/or honey characters in both control and flavored wines. In contrast, sensory differences observed between some control and flavored Shiraz wines, SH1+R in particular, persisted during bottle aging, such that the sensory impact of flavor additives on wine aroma and/or flavor was still apparent. These results demonstrate that flavorings could be used to influence the sensory profiles of wine, but the optimization of the concentration and composition of flavorings would be needed to achieve lasting sensory outcomes.

Although flavor additives are routinely used in many food and beverage industries, they are not legally permitted winemaking aids [4], so their use in wine is currently prohibited. It is unlikely that flavorings would ever be used in the production of premium quality wines, for which winemakers and consumers alike value traditional approaches to winemaking. However, this study demonstrates the potential for flavorings to be used to mitigate sensory deficiencies in lower quality and/or commodity wines, if the regulations governing winemaking additives were reviewed. For example, flavorings could be used to enhance the aroma and/or flavor of wines which lack intensity (e.g., due to adverse weather conditions, such as unusually cool seasons, or prolonged drought or heat), or to mask the presence of undesirable characters (e.g., green or earthy notes) or even mild faults or taints (e.g., reductive characters or mustiness). Given the current (global) trend towards producing wines of lower alcohol content [30], there might also be opportunities for flavor additives to be used in conjunction with various alcohol adjustment strategies, which can also affect wine aroma, flavor, and body. Additionally, flavorings could be used to introduce oak characters to wine (e.g., vanilla, coconut, or spice notes), without the investment in time or capital associated with traditional barrel maturation. Findings could also be applied in the production of wine products, i.e., wine made with the addition of flavorings, legally defined in Australia as ‘food containing no less than 700 mL/L of wine which has been formulated, processed, modified or mixed with other foods’ [4].

As indicated above, differences in the composition and/or performance of the DA panel between *t* = 0 and *t* = 1 are acknowledged as an inherent limitation of the study. The differences observed in the sensory profiles of control and flavored wines between time points may, in part, have been attributable to the DA panel. Nonetheless, significant differences were still observed between the sensory properties of some control and flavored wines at each time point. The DA panels identified several new attributes in bottle-aged wines, some of which were consistent with descriptors associated with bottle aging, i.e., dried fruit and toast for white wine and licorice and plum for red wine. Most importantly, there was no evidence to suggest that any chemical transformation of natural flavorings that might have occurred resulted in the formation of off-odors during bottle aging; i.e., at *t* = 1, the flavor additives had not negatively impacted wine sensory profiles.

## 4. Conclusions

The natural flavorings used in this study were found to contain volatile compounds previously identified in grapes and/or wine, but their addition to base wines did not always significantly impact wine composition; i.e., many of the volatile compounds identified as constituents of flavor additives were not detected at appreciably higher concentrations in flavored wines, which likely reflects the extremely small quantities of flavorings added to base wines. However, the addition of flavorings significantly modified the sensory profiles of wines, with flavored wines, CH1 + A, CH1 + PF, CH2 + PF, SH1 + R and SH2 + B in particular, exhibiting enhanced fruit and/or developed aromas and flavors, as a consequence of the use of flavor additives. In the case of Chardonnay wines, the variation in sensory properties resulting from the addition of flavorings diminished with time. However, the sensory impact arising from the addition of selected flavorings to Shiraz persisted after 12 months of bottle aging. This demonstrates the potential for flavor additives to be used to enhance desirable sensory attributes and/or mitigate sensory deficiencies.

## Figures and Tables

**Table 1:** Volatiles identified as abundant constituents of natural flavor additives by GC-MS analysis.

Flavor Additives	Volatile Compounds
apricot	linalool, hexyl butanoate
berry	linalool, *α*-terpineol, α-ionone
butter	ethyl butanoate
chocolate	2-ethyl-1-hexanol, ethyl butanoate
cinnamon	cinnamaldehyde, ethyl cinnamate, benzaldehyde
custard	ethyl butanoate
honey	2-phenylethyl acetate, ethyl acetate
oak	2-phenylethyl alcohol, furfural
orange	linalool, ethyl butanoate, limonene
passion fruit	2-phenethyl isovalerate, *cis*-3-hexenyl butyrate
raspberry	β-ionone, linalool
vanilla	vanillin, 2-ethyl-1-hexanol

**Table 2:** Peak areas for selected volatile constituents of control and flavored Chardonnay (CH1 and CH2) and Shiraz (SH1 and SH2) wines, after bottling (*t* = 0) and then after 12 months of bottle aging (*t* = 1).

Flavor Target	Compound	Wine Composition	
berry	linalool	SH2 (*t* = 0): 77,936 SH2 + B (*t* = 0): 120,887	SH2 (*t* = 1): 74,909 SH2 + B (*t* = 1): 110,252
	α-ionone	SH2 (*t* = 0): 1797 SH2 + B (*t* = 0): 35,769	SH2 (*t* = 1): 1842 SH2 + B (*t* = 1): 53,500
	β-ionone	SH2 (*t* = 0): 2686 SH2 + B (*t* = 0): 12,429	SH2 (*t* = 1): 2416 SH2 + B (*t* = 1): 27,276
chocolate	2-ethyl-1-hexanol	SH1 (*t* = 0): 693,470 SH1 + C (*t* = 0): 880,936	SH1 (*t* = 1): 903,129 SH1 + C (*t* = 1): 783,384
	linalool	SH1 (*t* = 0): 65,153 SH1 + C (*t* = 0): 67,702	SH1 (*t* = 1): 66,725 SH1 + C (*t* = 1): 430,975
	limonene	SH1 (*t* = 0): 14,347 SH1 + C (*t* = 0): 18,966	SH1 (*t* = 1): 18,458 SH1 + C (*t* = 1): 50,955
honey	2-phenylethyl acetate	CH2 (*t* = 0): 327,217 CH2 + H (*t* = 0): 734,763	CH2 (*t* = 1): 325,031 CH2 + H (*t* = 1): 511,441
passion fruit	*cis*-3-hexenyl butyrate	CH1 (*t* = 0): 4309 CH1 + PF (*t* = 0): 3232	CH1 (*t* = 1): 3272 CH1 + PF (*t* = 1): 87,350
		CH2 (*t* = 0): 3531 CH2 + PF (*t* = 0): 2990	CH2 (*t* = 1): 3400 CH2 + PF (*t* = 1): 161,211
	linalool	CH1 (*t* = 0): 46,231 CH1 + PF (*t* = 0): 43,152	CH1 (*t* = 1): 45,623 CH1 + PF (*t* = 1): 95,449
raspberry	linalool	SH1 (*t* = 0): 65,153 SH1 + R (*t* = 0): 62,545	SH1 (*t* = 1): 66,725 SH1 + R (*t* = 1): 282,633
	limonene	SH1 (*t* = 0): 14,347 SH1 + R (*t* = 0): 19,719	SH1 (*t* = 1): 18,458 SH1 + R (*t* = 1): 41,333

Peak areas were corrected against the internal standard (i.e., 2-octanol).

**Table 3:** Mean intensity ratings for aroma, flavor, taste, and mouthfeel attributes of control and flavored Chardonnay 1 (CH1) wines after bottling (*t* = 0) and then after 12 months of bottle aging (*t* = 1).

Attributes	CH1 *t* = 0	CH1 + A *t* = 0	CH1 + PF *t* = 0	*p*	CH1 *t* = 1	CH1 + A *t* = 1	CH1 + PF *t* = 1	*p*
Aroma								
passion fruit	7.4	7.4	8.9	ns	10.2	11.8	10.4	ns
tropical fruit	8.6	9.8	9.7	ns	9.5	9.4	9.0	ns
stone fruit	7.3 b	9.1 a	9.6 a	0.017	8.5	10.3	9.2	ns
citrus	3.6 b	6.3 a	5.8 a	0.001	6.7	6.3	5.9	ns
green	2.3	1.9	2.0	ns	2.9	1.5	1.0	ns
honey	5.1	5.3	4.1	ns	7.7	9.6	8.7	ns
vanilla	4.4	4.4	4.5	ns	7.5	10.5	8.1	ns
butter	3.8 a	3.2 a	2.1 b	0.002	8.0	9.3	8.6	ns
orange blossom	3.1 b	5.7 a	4.0 b	0.010	7.5	8.3	8.1	ns
dried stone fruit ^a^	-	-	-	-	8.5	10.6	10.3	ns
Melon ^a^	-	-	-	-	4.0 b	5.3 ab	6.5 a	0.009
Toast ^a^	-	-	-	-	6.4	6.9	5.8	ns
Flavor								
passion fruit	9.5	9.2	9.2	ns	11.1	10.6	9.6	ns
stone fruit	9.0	9.5	9.6	ns	10.2	9.4	10.1	ns
mixed spice	4.6 b	5.9 a	3.6 c	<0.001	8.8	8.2	8.8	ns
caramel-lolly	4.3 b	5.3 a	3.7 b	0.005	6.8 b	10.0 a	9.5 a	0.017
oak	3.3 b	4.8 a	3.3 b	0.013	8.7	9.9	9.1	ns
dried stone fruit ^a^	-	-	-	-	10.0	12.5	11.7	ns
green vegetable ^a^	-	-	-	-	3.1	1.9	2.7	ns
Taste and mouthfeel								
bitterness	5.5	5.6	5.2	ns	6.4	6.9	6.0	ns
acidity	8.0	7.9	8.3	ns	9.0	9.7	8.9	ns
astringency	6.1 a	3.9 c	4.9 b	<0.0001	8.4	7.1	7.2	ns
creaminess	5.8 a	3.8 b	4.0 b	<0.0001	6.3 b	9.5 a	7.5 ab	0.044
aftertaste	10.9 a	6.4 b	7.1 b	<0.0001	10.0	10.8	10.1	ns

Values are mean scores from 4 replicates per treatment, determined by 11 judges at *t* = 0 and from 3 replicates per treatment, determined by 12 judges at *t* = 1. Mean values followed by a different letter within a row (by treatment for each time point) are significantly different (*p* ≤ 0.05, one way ANOVA, Fisher’s LSD post hoc); ns = not significant. ^a^ Attributes associated with aged wines only.

**Table 4:** Mean intensity ratings for aroma, flavor, taste, and mouthfeel attributes of control and flavored Chardonnay 2 (CH2) wines after bottling (*t* = 0) and then after 12 months of bottle aging (*t* = 1).

Attributes	CH2 *t* = 0	CH2 + H *t* = 0	CH2 + PF *t* = 0	*p*	CH2 *t* = 1	CH2 + H *t* = 1	CH2 + PF *t* = 1	*p*
Aroma								
passion fruit	10.0 a	9.5 a	7.6 b	<0.001	11.7	10.4	11.3	ns
tropical fruit	10.5	10.5	10.1	ns	10.2	8.8	10.8	ns
stone fruit	6.5 ab	8.2 a	6.0 b	ns	8.0 b	8.9 ab	10.8 a	0.016
citrus	6.2 a	3.3 b	2.6 b	<0.0001	7.7	7.1	8.7	ns
green	1.7 a	1.0 b	1.0 b	0.012	3.3	2.1	3.0	ns
honey	5.1 a	5.2 a	3.2 b	0.029	6.1 b	9.5 a	7.3 b	0.010
vanilla	5.5	4.1	5.3	ns	7.0	8.9	6.5	ns
butter	3.5 a	2.9 a	1.7 b	<0.001	8.1	8.0	7.6	ns
orange blossom	2.4	2.1	2.7	ns	7.3	8.1	9.3	ns
dried stone fruit ^a^	-	-	-	-	8.4	10.2	8.3	ns
Melon ^a^	-	-	-	-	5.6	5.6	6.1	ns
Toast ^a^	-	-	-	-	5.2 ab	6.6 a	4.5 b	0.026
Flavor								
passion fruit	9.8	10.8	9.5	ns	12.0 a	10.1 b	12.1 a	0.036
stone fruit	7.7	8.8	7.1	ns	10.0	9.6	11.6	ns
mixed spice	2.5 b	3.3 b	2.4 b	ns	5.7	6.3	7.0	ns
caramel-lolly	3.1	3.5	3.2	ns	6.2	9.0	7.0	ns
oak	2.3 b	3.7 a	2.9 b	0.008	6.6 b	9.3 a	6.0 b	0.004
dried stone fruit ^a^	-	-	-	-	9.6	11.0	9.9	ns
green vegetable ^a^	-	-	-	-	3.5	2.0	4.1	ns
Taste and mouthfeel								
bitterness	3.0 c	5.3 b	9.7 a	<0.0001	6.1	4.8	4.9	ns
acidity	7.2 b	6.7 b	11.2 a	<0.0001	10.0	9.8	11.3	ns
astringency	5.4 b	5.0 b	9.3 a	<0.0001	6.6	6.6	7.4	ns
creaminess	7.3 ab	6.4 b	8.5 a	0.006	7.2	7.8	6.3	ns
aftertaste	7.9 b	9.6 ab	10.7 a	<0.001	8.9 b	11.2 a	10.0 ab	0.0003

Values are mean scores from 4 replicates per treatment, determined by 11 judges at *t* = 0 and from 3 replicates per treatment, determined by 12 judges at *t* = 1. Mean values followed by a different letter within a row (by treatment for each time point) are significantly different (*p* ≤ 0.05, one way ANOVA, Fisher’s LSD post hoc); ns = not significant. ^a^ Attributes associated with aged wines only.

**Table 5:** Mean intensity ratings for aroma, flavor, taste, and mouthfeel attributes of control and flavored Shiraz 1 (SH1) wines after bottling (*t* = 0) and then after 12 months of bottle aging (*t* = 1).

Attributes	SH1 *t* = 0	SH1 + C *t* = 0	SH1 + R *t* = 0	*p*	SH1 *t* = 1	SH1 + C *t* = 1	SH1 + R *t* = 1	*p*
Aroma								
red berry	5.2	6.4	6.1	ns	4.9	6.2	5.0	ns
dark berry	7.7	8.1	7.6	ns	6.9	6.4	7.1	ns
confectionary	5.4 b	5.8 b	8.3 a	<0.001	3.6 b	4.6 b	6.5 a	<0.0001
chocolate-vanilla	5.4 b	5.3 b	8.4 a	<0.0001	4.7 b	4.8 b	7.8 a	<0.0001
mixed spice	6.4	6.1	5.5	ns	4.4	4.8	4.9	ns
earthy	2.2 b	3.0 a	1.7 b	0.003	4.8	3.5	4.2	ns
green	2.1	2.8	1.6	ns	4.6 a	3.4 ab	2.5 b	0.006
black pepper	4.8	5.2	4.6	ns	5.7	4.8	4.3	ns
Plum ^a^	-	-	-	-	5.2	4.8	4.9	ns
Licorice ^a^	-	-	-	-	4.1	4.0	4.0	ns
dried herbs ^a^	-	-	-	-	3.5	3.0	3.5	ns
Flavor								
red berry	6.7	6.4	7.0	ns	6.5	6.3	6.7	ns
dark berry	9.2	8.7	9.3	ns	7.7	7.1	7.2	ns
confectionary	5.2 ab	4.8 b	6.6 a	0.043	4.4 b	5.1 b	6.5 a	0.003
mixed spice	6.5	6.0	6.3	ns	5.2 ab	4.7 b	6.0 a	0.021
chocolate-vanilla	4.2 b	3.9 b	6.4 a	<0.0001	5.0 b	5.2 b	7.1 a	<0.001
oak	7.4	7.0	7.9	ns	5.8 b	5.6 b	6.8 a	0.020
Cherry ^a^	-	-	-	-	6.4 a	4.8 b	6.8 a	0.013
green vegetable ^a^	-	-	-	-	4.2	3.4	2.8	ns
Taste and mouthfeel								
bitterness	6.5	6.1	5.2	0.006	5.9	6.3	4.8	0.045
acidity	7.4	7.9	7.5	ns	6.0	6.0	6.2	ns
astringency	7.9	7.8	8.4	ns	7.8	8.3	7.5	ns
alcohol	8.4	7.7	7.7	ns	7.3	7.4	6.6	0.040
length	10.6	10.4	10.9	ns	7.0	7.1	7.5	ns

Values are mean scores from 4 replicates per treatment, determined by 12 judges at *t* = 0 and from 3 replicates per treatment, determined by 12 judges at *t* = 1. Mean values followed by a different letter within a row (by treatment for each time point) are significantly different (*p* ≤ 0.05, one way ANOVA, Fisher’s LSD post hoc); ns = not significant. ^a^ Attributes associated with aged wines only.

**Table 6:** Mean intensity ratings for aroma, flavor, taste, and mouthfeel attributes of control and flavored Shiraz 2 (SH2) wines after bottling (*t* = 0) and then after 12 months of bottle aging (*t* = 1).

Attributes	SH2 *t* = 0	SH2 + B *t* = 0	SH2 + R *t* = 0	*p*	SH2 *t* = 1	SH2 + B *t* = 1	SH2 + R *t* = 1	*p*
Aroma								
red berry	6.3	7.0	6.8	ns	5.4 b	7.7 a	6.2 b	<0.001
dark berry	7.9	7.7	8.4	ns	6.0	6.6	7.1	ns
confectionary	6.4	8.0	6.7	ns	4.1 b	6.4 a	5.0 b	0.002
chocolate-vanilla	4.6	5.3	4.9	ns	3.9	5.2	4.4	ns
mixed spice	6.0	5.5	5.6	ns	3.6	3.7	4.9	ns
earthy	2.3	2.7	2.1	ns	4.7 a	3.0 b	4.2 a	0.013
green	2.4	2.6	2.8	ns	4.5	4.0	3.6	ns
black pepper	5.2	4.7	4.8	ns	4.3	5.3	4.5	ns
Plum ^a^	-	-	-	-	5.4	4.8	6.0	ns
Licorice ^a^	-	-	-	-	4.6	5.0	4.1	ns
dried herbs ^a^	-	-	-	-	3.1	2.9	2.9	ns
Flavor								
red berry	7.5	8.7	7.8	ns	7.3	8.2	7.7	ns
dark berry	9.2	9.8	9.7	ns	7.4	7.6	7.4	ns
confectionary	7.5 ab	8.8 a	6.7 b	0.035	6.6	7.1	7.1	ns
mixed spice	5.4	5.4	5.5	ns	4.1	5.2	5.3	ns
chocolate-vanilla	4.8	5.5	4.4	ns	4.3 b	5.8 a	4.9 ab	0.016
oak	8.2 a	6.2 b	7.6 a	0.002	4.9	4.9	4.9	ns
Cherry ^a^	-	-	-	-	6.7	6.9	4.9	ns
green vegetable ^a^	-	-	-	-	3.7	3.7	2.9	ns
Taste and mouthfeel								
bitterness	5.3	4.6	5.0	ns	5.3 a	4.4 b	4.4 b	0.050
acidity	6.8	7.3	7.3	ns	6.2	5.8	6.4	ns
astringency	7.9	7.9	7.9	ns	7.0	6.0	6.6	ns
alcohol	7.1 b	8.1 a	7.9 a	0.040	6.7	6.4	6.9	ns
length	9.8	10.3	10.1	ns	7.1	7.5	7.4	ns

Values are mean scores from 4 replicates per treatment, determined by 12 judges at *t* = 0 and from 3 replicates per treatment, determined by 12 judges at *t* = 1. Mean values followed by a different letter within a row (by treatment for each time point) are significantly different (*p* ≤ 0.05, one way ANOVA, Fisher’s LSD post hoc); ns = not significant. ^a^ Attributes associated with aged wines only.

## Supplementary Materials

The following are available online at https://www.mdpi.com/2304-8158/9/9/1208/s1, Table S1: Flavorings added to Chardonnay and Shiraz wines. Table S2: Aroma descriptors and GC-MS method characteristics (retention times and ions) of key constituents of flavor additives. Table S3: Attributes and standards used in the descriptive analysis of Chardonnay and Shiraz wines. Table S4: Analysis of variance *p* values for sensory attributes of Chardonnay 1 (CH1) wines after bottling (at *t* = 0) and after 12 months bottle aging (at *t* = 1). Table S5: Analysis of variance *p* values for sensory attributes of Chardonnay 2 (CH2) wines after bottling (at *t* = 0) and after 12 months bottle aging (at *t* = 1). Table S6: Analysis of variance *p* values for sensory attributes of Shiraz 1 (SH1) wines after bottling (at *t* = 0) and after 12 months bottle aging (at *t* = 1). Table S7: Analysis of variance *p* values for sensory attributes of Shiraz 2 (SH2) wines after bottling (at *t* = 0) and after 12 months bottle aging (at *t* = 1). Table S8: pH, titratable acidity (TA), alcohol, residual sugar, and volatile acidity (VA) of control and flavored Chardonnay and Shiraz wines after 12 months bottle aging (*t* = 1).
