# Supplementary material for: Impact of Bottle Aging on the Composition and Sensory Properties of Flavored Chardonnay and Shiraz Wines
Source: Foods. 2020 Sep 1;9(9):1208. doi: 10.3390/foods9091208 (PMC7555831; doi:10.3390/foods9091208)
Supplement: Supplementary file 1 [file foods-09-01208-s001.pdf]

foods

MDPI

Supplementary materials

Table S1. Flavorings added to Chardonnay (CH1 and CH2) and Shiraz (SH1 and SH2) wines.

Wine	Flavor Target	Wine Code	Flavor Additives
CH1	apricot	CH1 + A	1.8 g/L apricot[a], 0.6 g/L oak[a], 2.2 g/L butter[a]
	passion fruit	CH1 + PF	2.3 g/L passion fruit[a], 2.2 g/L butter[a], 0.5 g/L custard[b]
CH2	honey	CH2 + H	1.4 g/L honey[a], 1.5 g/L butter[a], 0.2 g/L vanilla[b]
	passion fruit	CH2 + PF	2.2 g/L passion fruit[a], 1.5 g/L butter[a]
SH1	chocolate	SH1 + C	3.0 g/L butter[a], 1.0 g/L cinnamon[b], 1.5 g/L orange[b], 2.9 g/L chocolate[b]
	raspberry	SH1 + R	3.0 g/L butter[a], 1.6 g/L orange[b], 2.2 g/L custard[b], 0.5 g/L raspberry[a]
SH2	berry	SH2 + B	1.7 g/L berry[a], 0.4 g/L custard[b], 1.8 g/L butter[a]
	raspberry	SH2 + R	0.5 g/L raspberry[a], 2.1 g/L butter[a]

[a] Flavor additive from FlavorSense Corporation. [b] Flavor additive from The Product Makers.

Table S2. Aroma descriptors and gas chromatography-mass spectrometry (GC-MS) method characteristics (retention times and ions) of key constituents of flavor additives.

Compound	Descriptors[a]	Retention Time (min)	Ions[b] (*m*/*z*)
benzaldehyde	almond, burnt sugar	42.9	**106**, 105, 77
cinnamaldehyde	cinnamon	71.4	132, **131**, 103
β-citronellol	rose	57.1	81, **69**, 41
ethyl acetate	nail polish	6.6	70, 61, **43**
ethyl butanoate	fruity	12.3	88, **71**, 43
ethyl cinnamate	strawberry cream	75.4	176, **131**, 103
2-ethyl hexanol	floral, fruity	41.0	83, **57**, 41
furfural	earthy, wood	39.5	**96**, 95, 39
cis-3-hexenyl butyrate	green apple, fruity	39.3	82, 71, **67**
hexyl butanoate	apple peel	36.3	89, **71**, 43
α-ionone	sweet fruit	61.3	136, **121**, 93
β-ionone	violets	65.9	**177**, 135, 43
linalool	floral, citrus	44.6	121, 93, **71**
phenethyl acetate	floral, rose, fruity	59.7	**104**, 91, 43
phenethyl alcohol	floral, rose	64.6	122, 92, **91**
phenethyl isovalerate	fruity, pineapple	68.7	105, **104**, 57
α-terpineol	spicy	53.1	121, 93, **59**
vanillin	vanilla	94.3	152, **151**, 81

[a] Sourced from [23,30] and references therein. [b] Ions in bold were used for quantification.

Table S3. Attributes and standards used in descriptive analysis of Chardonnay and Shiraz wines.

Attribute	Reference Standard
White wine descriptors	
passion fruit	4 drops passion fruit flavor additive[a]
tropical fruit	1 cm^3 each of paw paw + pineapple + mango + melon
stone fruit	1 cm^3 each of white peach + nectarine, 4 drops apricot flavor additive[a]
citrus	1 cm^3 each of mandarin + lemon + orange
green	0.15 g freshly cut grass
honey	½ tbsp of honey (Capilano)
vanilla	3 drops vanilla flavor additive[b]
butter	5 drops butter flavor additive[b]
orange blossom	0.3 g freshly cut leaves from an orange tree
mixed spice	¼ tsp of allspice (McKenzies) + 4 drops cinnamon flavor additive[b]
caramel lolly	1 caramel lolly (Coles brand) cut into small pieces
oak	0.07 g medium toasted American oak chips (O.C. Inc.)
dried stone fruit	½ dried apricot + ½ dried peach cut into small pieces
melon	1 cm^3 of honeydew melon
toast	2 toasted almonds crushed (Woolworths Select)
green vegetable	1 cm^3 each of green apple + green capsicum
Red wine descriptors	
red berry	2 frozen raspberries + 1 frozen strawberry (McCains)
dark berry	5 drops blackcurrant flavor additive[b] + 6 drops blackberry flavor additive[b]
confectionary	6 drops raspberry flavor additive[a] + 6 drops berry flavor additive[a]
chocolate-vanilla	3 drops vanilla flavor additive[b] + 10 drops chocolate flavor additive[b]
mixed spice	6 drops cinnamon flavor additive[b]
earthy	30 g wet earth
green	2 frozen blackcurrants + 1 frozen blackberry (McCains)
black pepper	0.02 g black pepper (McCormick)
oak	0.07 g medium toasted American oak chips (O.C. Inc.)
plum	1 plum (20 g) cut into small pieces (Coles brand)
licorice	licorice (6 g) cut into small pieces (Coles brand)
dried herbs	0.02 g oregano (McCormick) + 0.02 g thyme (McCormick)
cherry	1 pitted sour cherry (3–5 g) (Always Fresh)
green vegetable	1 cm^3 each of green apple + green capsicum
Taste and mouthfeel descriptors	
bitterness	quinine sulfate (low 5 mg/L – high 20 mg/L)
acidity	tartaric acid (low 0.5 g/L – high 2g/L)
astringency	felt material (low) – sandpaper (high)
creaminess	low fat milk (low) to full cream milk (high)

Standards were prepared in 30 mL of unoaked Chardonnay or Shiraz cask wine. [a] Flavor additives from FlavorSense Corporation. [b] Flavor additives from The Product Makers.

Table S4. Analysis of variance *p* values for sensory attributes of Chardonnay 1 (CH1) wines after bottling (at $t = 0$) and after 12 months bottle aging (at $t = 1$).

Attributes	Wine	Panelist	Replicate	Wine x Panelist	Wine x Replicate	Panelist x Replicate	Wine	Panelist	Replicate	Wine x Panelist	Wine x Replicate	Panelist x Replicate
Aroma												
passion fruit	0.085	0.539	0.771	0.063	0.003	0.070	0.124	< 0.001	0.135	0.022	0.406	0.023
tropical fruit	0.145	< 0.0001	0.445	0.240	0.003	0.106	0.869	< 0.0001	0.969	0.847	0.029	0.310
stone fruit	0.017	< 0.001	0.273	0.109	0.181	0.056	0.228	< 0.0001	0.801	0.206	0.469	0.838
citrus	0.001	< 0.0001	0.135	0.235	< 0.001	0.043	0.634	< 0.0001	0.627	0.183	0.110	0.158
green	0.811	< 0.0001	0.003	0.032	< 0.0001	0.010	0.056	0.012	0.314	0.829	0.363	0.165
honey	0.171	< 0.0001	0.004	0.456	0.556	0.469	0.174	< 0.0001	0.172	0.867	0.107	0.352
vanilla	0.998	< 0.0001	< 0.001	0.573	0.608	0.023	0.065	< 0.001	0.970	0.970	0.711	1.000
butter	0.002	< 0.0001	0.001	0.401	< 0.001	0.039	0.557	< 0.001	0.707	0.436	0.034	0.360
orange blossom	0.010	0.010	0.759	0.215	0.014	0.931	0.639	< 0.0001	0.480	0.313	0.119	0.183
dried stone fruit [a]	-	-	-	-	-	-	0.105	< 0.0001	0.716	0.222	0.199	0.281
melon[a]	-	-	-	-	-	-	0.009	< 0.0001	0.781	0.078	0.784	0.001
toast[a]	-	-	-	-	-	-	0.684	< 0.0001	0.663	0.824	0.211	0.468
Flavor												
passion fruit	0.692	< 0.0001	0.051	0.807	0.039	0.047	0.317	< 0.001	0.430	0.325	0.758	0.301
stone fruit	0.745	< 0.0001	0.068	0.659	0.002	0.404	0.642	< 0.0001	0.528	0.766	0.143	0.435
mixed spice	< 0.001	< 0.0001	0.002	0.010	< 0.0001	0.090	0.829	< 0.0001	0.465	0.362	0.929	0.687
caramel lolly	0.005	< 0.0001	< 0.001	0.684	< 0.001	0.364	0.017	0.003	0.397	0.363	0.966	0.594
oak	0.013	< 0.001	0.001	0.683	0.013	0.453	0.555	< 0.0001	0.935	0.472	0.734	0.532
dried stone fruit[a]	-	-	-	-	-	-	0.078	0.035	0.827	0.411	0.984	0.681
green vegetable[a]	-	-	-	-	-	-	0.310	< 0.0001	0.320	0.429	0.428	0.105
Taste and mouthfeel												
bitterness	0.876	< 0.001	< 0.001	0.679	0.410	0.016	0.736	< 0.0001	0.676	0.075	0.814	0.982
acidity	0.647	< 0.0001	0.543	0.438	0.706	0.433	0.717	0.018	0.291	0.630	0.975	0.909
astringency	< 0.0001	< 0.0001	< 0.001	0.015	< 0.0001	0.004	0.172	< 0.0001	0.142	0.048	0.635	0.011
creaminess	< 0.0001	< 0.0001	< 0.0001	0.002	< 0.001	< 0.0001	0.044	0.002	0.991	0.922	0.836	0.823
aftertaste	< 0.0001	< 0.0001	0.002	0.125	< 0.0001	< 0.0001	0.421	< 0.0001	0.153	0.556	0.709	0.650

Significance was $p < 0.05$. Attributes associated with aged wines only.

Table S5. Analysis of variance *p* values for sensory attributes of Chardonnay 2 (CH2) wines after bottling (at $t = 0$) and after 12 months bottle aging (at $t = 1$).

Attributes	Wine	Panelist	Replicate	Wine x Panelist	Wine x Replicate	Panelist x Replicate	Wine	Panelist	Replicate	Wine x Panelist	Wine x Replicate	Panelist x Replicate
Aroma												
passion fruit	< 0.001	< 0.0001	< 0.0001	0.731	< 0.001	< 0.001	0.255	< 0.0001	0.647	0.011	0.299	0.768
tropical fruit	0.797	< 0.0001	0.001	0.010	0.197	< 0.001	0.116	< 0.0001	0.744	0.909	0.295	0.667
stone fruit	0.061	< 0.0001	0.232	0.891	0.033	0.403	0.016	< 0.0001	0.462	0.885	0.168	0.332
citrus	< 0.0001	0.003	0.267	0.111	0.041	0.003	0.314	< 0.0001	0.280	0.427	0.750	0.087
green	0.012	< 0.0001	< 0.0001	0.101	< 0.0001	< 0.0001	0.505	0.002	0.441	0.843	0.974	0.645
honey	0.029	< 0.0001	0.086	0.015	0.602	0.161	0.010	< 0.0001	0.083	0.289	0.588	0.927
vanilla	0.214	< 0.0001	0.098	0.569	0.003	0.195	0.094	< 0.0001	0.091	0.101	0.333	0.671
butter	< 0.001	< 0.0001	0.027	0.647	0.044	0.001	0.890	< 0.0001	0.031	0.767	0.106	0.367
orange blossom	0.539	< 0.0001	0.006	0.175	0.226	0.055	0.166	< 0.0001	0.150	0.664	0.636	0.444
dried stone fruit [a]	-	-	-	-	-	-	0.130	< 0.0001	0.982	0.031	0.370	0.825
melon[a]	-	-	-	-	-	-	0.882	< 0.001	0.433	0.104	0.259	0.434
toast[a]	-	-	-	-	-	-	0.026	< 0.0001	0.228	0.001	0.896	0.162
Flavo*r*												
passion fruit	0.160	< 0.001	0.262	0.373	0.373	0.040	0.036	< 0.0001	0.427	0.394	0.882	0.123
stone fruit	0.246	0.019	0.688	0.681	0.029	0.390	0.103	< 0.0001	0.090	0.820	0.925	0.221
mixed spice	0.052	< 0.0001	0.006	< 0.0001	0.013	< 0.0001	0.377	< 0.0001	0.950	0.371	0.036	0.387
caramel lolly	0.373	< 0.0001	< 0.0001	0.125	0.070	< 0.0001	0.068	< 0.0001	0.767	0.354	0.574	0.947
oak	0.008	< 0.0001	0.214	0.029	0.041	< 0.001	0.004	< 0.0001	0.267	0.049	0.609	0.513
dried stone fruit[a]	-	-	-	-	-	-	0.347	< 0.001	0.892	0.030	0.090	0.168
green vegetable[a]	-	-	-	-	-	-	0.055	< 0.0001	0.890	0.094	0.457	0.085
Taste and mouthfeel												
bitterness	< 0.0001	0.001	0.006	0.007	< 0.0001	0.067	0.306	< 0.0001	0.247	0.733	0.202	0.324
acidity	< 0.0001	0.001	0.963	< 0.001	< 0.0001	0.015	0.233	< 0.0001	0.889	0.547	0.520	0.956
astringency	< 0.0001	< 0.0001	0.079	0.036	< 0.0001	0.474	0.421	< 0.0001	0.006	0.013	0.443	0.243
creaminess	0.006	0.003	< 0.0001	0.304	0.102	0.011	0.415	< 0.0001	0.265	0.697	0.665	0.310
aftertaste	< 0.001	< 0.0001	0.002	0.039	0.003	0.004	0.003	< 0.0001	0.735	0.113	0.848	0.177

Significance was $p < 0.05$. Attributes associated with aged wines only.

Table S6. Analysis of variance *p* values for sensory attributes of Shiraz 1 (SH1) wines after bottling (at $t = 0$) and after 12 months bottle aging (at $t = 1$).

Attributes	Wine	Panelist	Replicate	Wine x Panelist	Wine x Replicate	Panelist x Replicate	Wine	Panelist	Replicate	Wine x Panelist	Wine x Replicate	Panelist x Replicate
Aroma												
red berry	0.090	< 0.0001	0.683	0.391	0.278	< 0.001	0.141	0.002	0.454	0.054	0.151	0.704
dark berry	0.697	< 0.0001	0.464	< 0.001	0.915	0.009	0.424	< 0.0001	0.762	0.152	0.422	0.249
confectionary	< 0.001	< 0.0001	0.011	0.310	0.945	0.008	< 0.0001	< 0.0001	0.317	< 0.001	0.320	0.233
chocolate-vanilla	< 0.0001	< 0.0001	0.141	0.196	0.329	0.018	< 0.0001	< 0.0001	0.087	0.008	0.806	0.282
mixed spice	0.232	< 0.0001	0.908	0.719	0.079	0.845	0.686	< 0.0001	0.284	0.139	0.337	0.134
earthy	0.003	< 0.0001	0.005	0.009	0.137	0.010	0.219	< 0.001	0.578	0.304	0.809	0.667
green	0.070	< 0.0001	0.343	0.914	0.861	0.588	0.006	< 0.0001	0.811	0.016	0.845	0.432
black pepper	0.540	< 0.0001	0.809	0.387	0.318	0.774	0.072	< 0.0001	0.428	0.539	0.329	0.282
plum[a]	-	-	-	-	-	-	0.896	< 0.001	0.964	0.876	0.688	0.997
licorice[a]	-	-	-	-	-	-	0.982	< 0.0001	0.559	0.087	0.430	0.125
dried herbs[a]	-	-	-	-	-	-	0.479	< 0.0001	0.717	0.015	0.429	0.970
Flavor												
red berry	0.622	< 0.0001	0.803	0.251	0.036	0.290	0.810	0.001	0.631	0.226	0.191	0.948
dark berry	0.466	< 0.0001	0.248	0.024	0.676	0.582	0.590	0.001	0.689	0.397	0.301	0.794
confectionary	0.043	< 0.001	0.751	0.728	0.244	0.013	0.003	< 0.0001	0.622	0.002	0.063	0.209
mixed spice	0.703	< 0.0001	0.410	0.045	0.997	0.054	0.021	< 0.0001	0.087	0.212	0.071	0.444
chocolate-vanilla	< 0.0001	< 0.0001	0.949	0.075	0.863	0.019	< 0.001	< 0.0001	0.217	0.023	0.012	0.637
oak	0.259	< 0.0001	0.052	0.493	0.620	0.010	0.020	< 0.0001	0.028	0.005	0.007	0.493
Cherry [a]	-	-	-	-	-	-	0.013	< 0.0001	0.768	0.557	0.993	0.999
green vegetable[a]	-	-	-	-	-	-	0.070	< 0.001	0.429	0.009	0.208	0.400
Taste and mouthfeel												
bitterness	0.006	< 0.0001	0.338	0.041	0.101	0.066	0.045	< 0.0001	0.727	0.753	0.300	0.504
acidity	0.621	< 0.0001	0.975	0.920	0.857	0.398	0.844	< 0.0001	0.550	0.861	0.368	0.004
astringency	0.487	< 0.0001	0.433	0.978	0.850	0.249	0.079	< 0.0001	0.533	0.069	0.137	0.767
alcohol	0.248	< 0.0001	0.379	0.297	0.534	0.092	0.105	< 0.0001	0.294	0.104	0.489	0.302
length	0.749	< 0.0001	0.946	0.882	0.150	0.998	0.326	< 0.0001	0.148	0.579	0.624	0.613

Significance was $p < 0.05$. Attributes associated with aged wines only.

Table S7. Analysis of variance *p* values for sensory attributes of Shiraz 2 (SH2) wines after bottling (at $t = 0$) and after 12 months bottle aging (at $t = 1$).

Attributes	Wine	Panelist	Replicate	Wine x Panelist	Wine x Replicate	Panelist x Replicate	Wine	Panelist	Replicate	Wine x Panelist	Wine x Replicate	Panelist x Replicate
Aroma												
red berry	0.610	< 0.0001	0.766	0.608	0.342	0.909	0.000	< 0.0001	0.491	0.068	0.881	0.037
dark berry	0.526	< 0.0001	0.438	0.841	0.379	0.239	0.153	< 0.0001	0.274	0.359	0.215	0.244
confectionary	0.199	0.001	0.352	0.887	0.924	0.849	0.002	< 0.0001	0.645	0.385	0.148	0.608
chocolate-vanilla	0.465	< 0.0001	0.963	0.331	0.725	0.026	0.158	< 0.0001	0.382	0.246	0.530	0.439
mixed spice	0.634	< 0.0001	0.615	0.031	0.166	0.003	0.112	< 0.0001	0.654	0.726	0.354	0.424
earthy	0.422	< 0.0001	0.635	0.003	0.038	0.957	0.013	< 0.0001	0.177	0.001	0.666	0.637
green	0.624	< 0.0001	0.004	0.151	0.561	0.003	0.483	< 0.0001	0.651	0.790	0.431	0.701
black pepper	0.671	< 0.0001	0.738	0.640	0.163	0.595	0.238	< 0.0001	0.595	0.263	0.124	0.916
plum [a]	-	-	-	-	-	-	0.252	< 0.0001	0.745	0.657	0.912	0.952
licorice[a]	-	-	-	-	-	-	0.244	< 0.0001	0.126	0.156	0.813	0.615
dried herbs[a]	-	-	-	-	-	-	0.903	< 0.0001	0.794	0.675	0.161	0.599
Flavor												
red berry	0.258	< 0.0001	0.894	0.861	0.881	0.936	0.188	0.000	0.742	0.200	0.889	0.277
dark berry	0.615	< 0.0001	0.845	0.854	0.652	0.061	0.864	< 0.0001	0.772	0.118	0.429	0.637
confectionary	0.035	0.003	0.466	0.440	0.485	0.707	0.413	< 0.0001	0.421	0.276	0.440	0.618
mixed spice	0.974	< 0.0001	0.508	0.488	0.614	< 0.001	0.105	< 0.0001	0.169	0.674	0.743	0.397
chocolate-vanilla	0.173	< 0.0001	0.048	0.066	0.726	0.413	0.016	< 0.0001	0.257	0.389	0.699	0.203
oak	0.002	< 0.0001	0.446	0.670	0.589	< 0.0001	0.998	0.000	0.907	0.965	0.905	0.871
cherry[a]	-	-	-	-	-	-	0.665	< 0.0001	0.290	0.214	0.822	0.006
green vegetable[a]	-	-	-	-	-	-	0.479	0.001	0.623	0.804	0.553	0.948
Taste and mouthfeel												
bitterness	0.361	< 0.0001	0.416	0.937	0.256	0.359	0.050	< 0.0001	0.031	0.002	0.493	0.175
acidity	0.568	< 0.0001	0.198	0.930	0.626	0.363	0.485	< 0.0001	0.066	0.898	0.329	0.895
astringency	0.997	< 0.0001	0.119	0.646	0.111	0.064	0.209	< 0.0001	0.069	0.778	0.563	0.868
alcohol	0.040	< 0.0001	0.114	0.064	0.660	0.002	0.499	< 0.0001	0.210	0.886	0.168	0.989
length	0.725	< 0.0001	0.006	0.455	0.013	0.020	0.409	< 0.0001	0.240	0.665	0.291	0.866

Significance was $p < 0.05$. Attributes associated with aged wines only.

Table S8. pH, titratable acidity (TA), alcohol, residual sugar and volatile acidity (VA) of control and flavored Chardonnay and Shiraz wines after 12 months bottle aging ($t = 1$).

**Wine**	**pH**	**TA (g/L)**	**Alcohol (% v/v)**	**Sugar (g/L)**	**VA (g/L)**
CH1	3.5	6.1	12.9	4.7	0.2
CH1+A	3.5	6.2	12.9	4.6	0.2
CH1+PF	3.5	6.2	12.9	4.6	0.2
CH2	3.4	6.6	12.0	2.6	0.3
CH2+H	3.4	6.6	12.0	2.7	0.3
CH2+PF	3.4	6.6	12.0	2.6	0.3
SH1	3.6	6.2	13.8	0.5	0.5
SH1+C	3.6	6.1	13.9	0.5	0.5
SH1+R	3.6	6.1	13.9	0.5	0.5
SH2	3.6	5.9	13.2	4.6	0.5
SH2+B	3.6	5.9	13.2	4.6	0.5
SH2+R	3.6	5.8	13.3	4.5	0.5

Values are means of two replicates. TA measured as g/L of tartaric acid; residual sugar measured as g/L of glucose and fructose; VA measured as g/L of acetic acid. No significant differences were observed between control and flavored wines ($p \leq 0.05$, one way ANOVA).
